# Causality in the Association between P300 and Alpha Event-Related Desynchronization

**DOI:** 10.1371/journal.pone.0034163

**Published:** 2012-04-12

**Authors:** Weiwei Peng, Li Hu, Zhiguo Zhang, Yong Hu

**Affiliations:** 1 Department of Orthopaedics and Traumatology, The University of Hong Kong, Hong Kong, China; 2 Key Laboratory of Cognition and Personality, Ministry of Education, and School of Psychology, Southwest University, Chongqing, China; 3 Department of Electrical and Electronic Engineering, The University of Hong Kong, Hong Kong, China; University Medical Center Groningen UMCG, The Netherlands

## Abstract

Recent findings indicated that both P300 and alpha event-related desynchronization (α-ERD) were associated, and similarly involved in cognitive brain functioning, e.g., attention allocation and memory updating. However, an explicit causal influence between the neural generators of P300 and α-ERD has not yet been investigated. In the present study, using an oddball task paradigm, we assessed the task effect (target vs. non-target) on P300 and α-ERD elicited by stimuli of four sensory modalities, i.e., audition, vision, somatosensory, and pain, estimated their respective neural generators, and investigated the information flow among their neural generators using time-varying effective connectivity in the target condition. Across sensory modalities, the scalp topographies of P300 and α-ERD were similar and respectively maximal at parietal and occipital regions in the target condition. Source analysis revealed that P300 and α-ERD were mainly generated from posterior cingulate cortex and occipital lobe respectively. As revealed by time-varying effective connectivity, the cortical information was consistently flowed from α-ERD sources to P300 sources in the target condition for all four sensory modalities. All these findings showed that P300 in the target condition is modulated by the changes of α-ERD, which would be useful to explore neural mechanism of cognitive information processing in the human brain.

## Introduction

P300 is an important event-related potential (ERP) component elicited by infrequent and task-relevant stimulus, and it reflects the processes of attention, stimulus classification, and memory updating [1], [2], [3], [4]. Although P300 is extensively used to study the neural functions of perceptual and cognitive processes in a wide variety of basic and clinical applications [1], [5], [6], its neural generators are still not very clearly characterized. Several inconsistently reported brain regions responsible for the generation of P300 include frontal lobe, globus pallidus, temporal-parietal junction, posterior cingulate gyrus, parietal cortex, and medial temporal lobe [7], [8], [9], [10].

Recently, the study of electrophysiological brain oscillations has opened a new window toward the understanding of neural functions [11]. Changes of ongoing electroencephalography (EEG) activities in response to stimulus presentation may appear either as a transient increase (event-related synchronization [ERS]) or a transient decrease (event-related desynchronization [ERD]) of the power of EEG oscillations in specific frequency ranges [12]. Among them, a significant alpha-band (8–13 Hz in frequency) ERD (α-ERD) could be induced by both sensory stimulation (external event) across stimulus modalities [13], [14], [15] and cognitive processing (internal event) in various attention and memory tasks [16], [17], [18], [19]. For this reason, some studies showed that α-ERD was mainly related to sensory perception and judgment (modality dependent), and dominantly generated from the primary sensory cortices [16], [19], [20], whereas some other studies reported that α-ERD was accompanied with cognitive operations, and commonly maximal at the occipital regions regardless of the stimulus modality (modality independent) [21], [22].

Previously, both P300 and α-ERD have been consistently triggered by the target stimuli in the oddball task paradigm, and P300 was showed to be functionally associated with the cognitive processing reflected by α-ERD [15], [23]. Note that the investigation on the relationship between ERPs and ERDs showed a comprehensive and systematic view of cortical processing related to sensory stimuli [6], [15], [24]. However, there is a debate of the causal influence between the neural generators of P300 and α-ERD. While Yordanova et al [15] showed that α-ERD was guided by the internal events indexed by P300, Polich [3] reported that the latency and amplitude of P300 could stem from α-ERD.

In order to assess (1) the neural generators of P300 and α-ERD triggered by internal event and (2) their causal influence, we performed an oddball task paradigm with sensory stimuli of four modalities, i.e., audition, vision, somatosensory, and pain. The neural generators of P300 were estimated using distributed source analysis [25], and the sources of α-ERD induced by internal events were estimated using lead field weighted minimum norm (WMN) algorithm [26]. Following, the causal relationship between the neural generators of P300 and α-ERD was assessed using a Kalman smoother based time-varying effective connectivity inference method [27].

## Results

### Behavioral results

**Figure 1:**
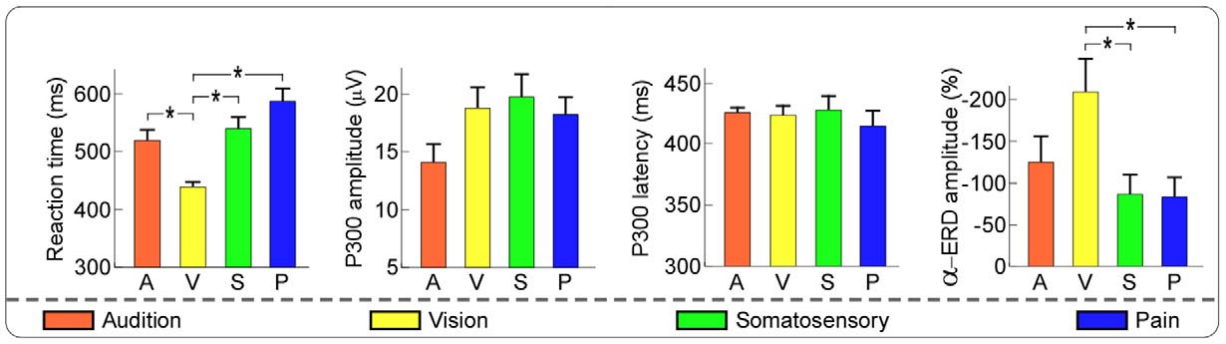
Comparison of reaction time, P300 latency, P300 amplitude, and α-ERD magnitude among all sensory modalities in target condition. Values are displayed in orange, yellow, green, and blue for auditory, visual, somatosensory, and pain target conditions respectively. Error bars represent, for each condition, ±SEM across subjects. Asterisk * indicates a significant difference (P<0.05).

The average values (mean ± SEM, the same hereinafter) of reaction time to the target stimuli were summarized in Table 1 and Fig. 1. Mauchly's test revealed that the assumption of sphericity had not been violated (chi-square = 4.26, P = 0.51), which indicated that there was no need to correct degrees of freedom. As revealed by 4-level (audition, vision, somatosensory, and pain) one-way repeated-measures analysis of variance (ANOVA), the reaction times were significantly different across sensory modalities (F (3, 51) = 29.42, P<0.001, partial Eta squared = 0.41). Post hoc tests revealed that reaction times to visual target stimuli were significantly shorter than those to auditory, somatosensory, and pain target stimuli (P<0.001 for all comparisons).

**Table 1:** Reaction time, P300 latency and amplitude, α-ERD magnitude in the target condition, and P200 latency and amplitude, α-ERD magnitude in the non-target condition.

	Parameters(mean ± SEM)	Sensory modalities			
		Audition	Vision	Somatosensory	Pain
**Target**	Reaction time (ms)	519±18	439±9	539±21	586±23
	P300 latency (ms)	425±6	423±9	427±13	413±13
	P300 amplitude (µV)	14.11±1.64	18.80±1.84	19.76±2.03	18.25±1.53
	α-ERD (ER%)	−124±32	−209±41	−87±24	−83±24
**Non-target**	P200 latency (ms)	254±5	401±8	339±14	345±14
	P200 amplitude (µV)	4.79±0.59	9.41±1.17	12.19±0.88	11.51±1.04
	α-ERD (ER%)	−38±11	−162±36	−44±12	−56±14

### Electrophysiological results

#### Time-Domain

Across subjects, latencies and amplitudes of P300 peak to the target stimuli and of P200 peak to the non-target stimuli were summarized in Table 1 and displayed in Fig. 1. The peak latencies of P200 were significantly shorter than those of P300 across the sensory modalities (P<0.001, paired sample t-test).

**Figure 2:**
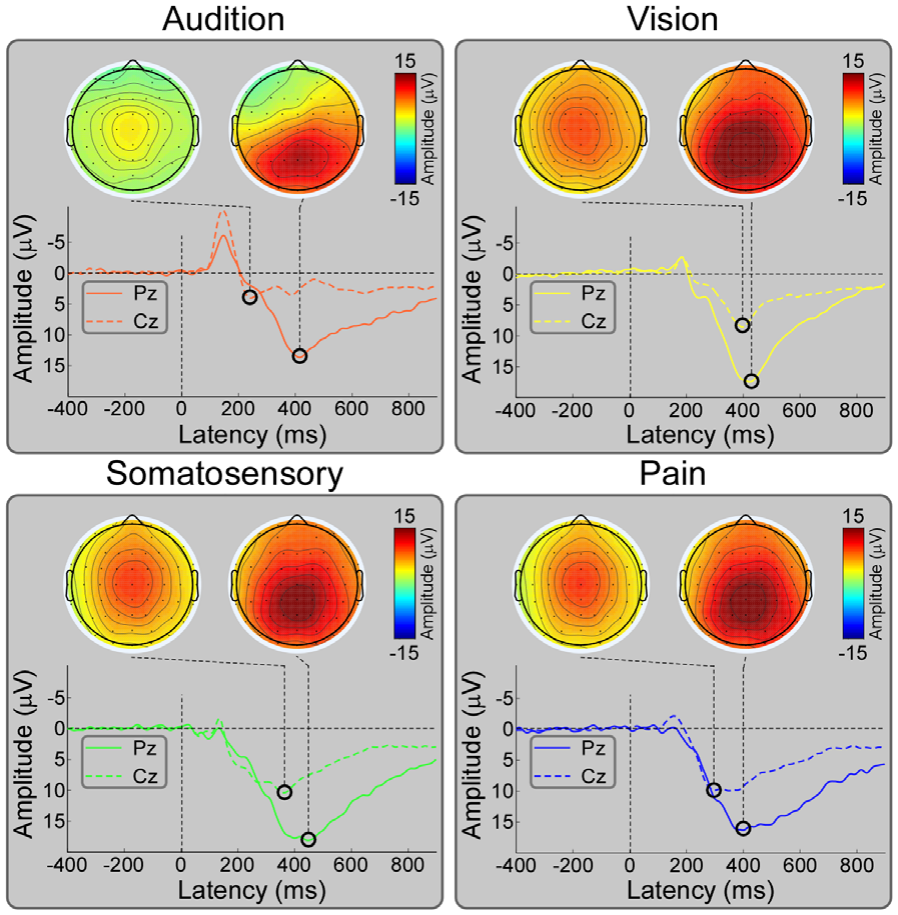
Grand average ERPs and scalp topographies of P200 and P300 for all sensory modalities in target and non-target conditions. Grand average ERP waveforms are measured at Pz in the target condition, and at Cz in the non-target condition across all sensory modalities. X-axis, latency (ms); Y-axis, amplitude (µV). Grand average ERP waveforms evoked by auditory, visual, somatosensory, and pain stimuli are presented in orange, yellow, green, and blue respectively. Noteworthy is that the scalp topographies of P300 elicited by the target stimuli across all sensory modalities were remarkably similar, and displayed a clear maximum at the parietal electrodes (around Pz). The scalp topographies of P200 elicited by the non-target stimuli across all sensory modalities were also remarkably similar, and displayed a clear maximum at the central electrodes (around Cz).

Fig. 2 showed the grand average ERP waveforms measured at Pz in the target conditions, and those measured at Cz in the non-target conditions. The scalp topographies of P300 in the target conditions and of P200 in the non-target conditions at their corresponding peak latencies were displayed. The scalp topographies of the P300 evoked by the auditory, visual, somatosensory, and pain target stimuli were remarkably similar, and displayed a clear maximum on the parietal region (around Pz). The scalp topographies of the P200 evoked by the auditory, visual, somatosensory, and pain non-target stimuli were also markedly similar, and displayed a clear maximum on the central region (around Cz).

#### Time-frequency domain

Across subjects, the magnitudes of α-ERD within the predefined ROI (target: 8–13 Hz in frequency and 300–800 ms in latency; non-target: 8–13 Hz in frequency and 200–700 ms in latency) in the target and non-target conditions were summarized in Table 1 and displayed in Fig. 1. Mauchly's test indicated that the assumption of sphericity had been violated (chi-square = 16.26, P<0.05), therefore degrees of freedom were corrected using Greenhouse-Geisser estimates of sphericity (epsilon = 0.59). As revealed by 4-level (audition vision, somatosensory, and pain) one-way repeated-measures ANOVA, the magnitudes of α-ERD in the target condition were significantly different across sensory modalities (F (1.78, 30.28) = 11.97, P = 0.006, partial Eta squared = 0.41). Post hoc tests revealed that α-ERD magnitudes induced by visual target stimuli were significantly higher than those induced by somatosensory and pain target stimuli (P = 0.009, P = 0.004, respectively) (Fig. 1). Note that the magnitudes of α-ERD in the target conditions were significantly higher than those in the non-target conditions across the sensory modalities (P<0.001, paired sample t-test).

**Figure 3:**
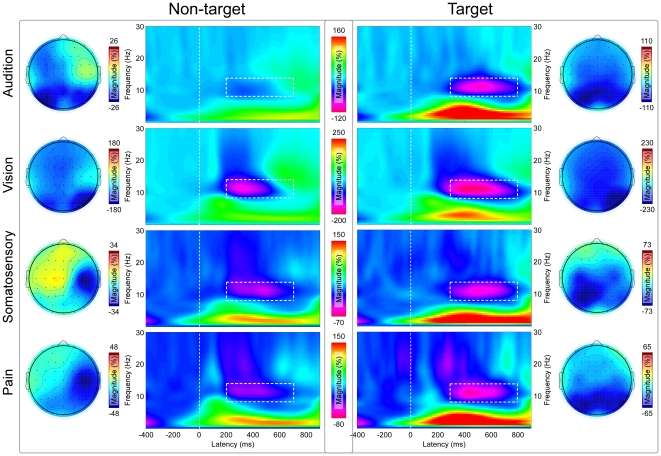
Grand average TFDs and α-ERD scalp topographies for all sensory modalities in target and non-target conditions. In the target condition, grand average TFDs are measured at (P3+P4+P5+P6+PO3+PO4)/6 for all sensory modalities, while, in the non-target condition, grand average TFDs are measured at (PO3+PO4+PO7+PO8+O1+O2)/6 for auditory and visual modalities and at (C4+C6+CP4)/3 for somatosensory and pain modalities. X-axis, latency (ms); Y-axis, frequency (Hz). Color scale represents baseline corrected oscillatory magnitude (ER%). It should be noted that the α-ERD induced by target stimuli is significantly larger in intensity, greater in size, and later in latency than that induced by non-target stimuli. The scalp topographies for “top 20%” magnitudes of α-ERD within the predefined ROI (marked using white rectangles) displayed a clear maximum at occipital regions across all sensory modalities in the target conditions, while, in the non-target conditions, they showed a clear maximum at occipital regions for auditory and visual modalities, and at contralateral central regions for somatosensory and pain modalities.

Fig. 3 showed the grand average TFDs and the corresponding scalp topographies for all four modalities in both the target and non-target conditions. It should be noted that the scalp topographies of α-ERD for all four modalities in target conditions displayed remarkably similar maximum in the occipital regions (around PO3 and PO4), whereas in the non-target conditions, they showed a maximum distribution at occipital regions only for auditory and visual modalities, but at contralateral central region for pain and somatosensory modalities (Fig. 3).

### Source analysis

**Figure 4:**
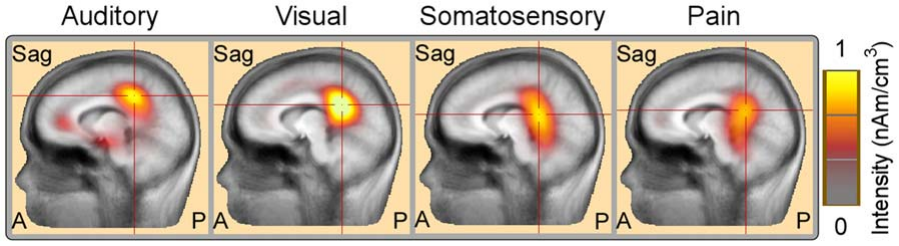
Source localizations of P300 elicited by target stimuli across all sensory modalities. Distributed sources estimated around P300 peak latencies using CLARA are superimposed on standard MR image template, and the color is coded according to their intensity, expressed in nAm/cm^3^. Talairach coordinates (x, y, z) of the sources of P300 are -9, -41, 37 mm; -9, -47, 19 mm; -9, -41, 13 mm; and -9, -47, 25 mm for auditory, visual, somatosensory, and pain target conditions respectively. Note that the sources of the P300 elicited by target stimuli across all sensory modalities are similarly located at the posterior cingulate cortex.

Fig. 4 showed the estimated sources of P300 evoked by auditory, visual, somatosensory, and pain target stimuli. The P300 sources in all target conditions were similarly located at the posterior cingulate cortex (Talairach coordinates: -9, -41, 37 mm; -9, -47, 19 mm; -9, -41, 13 mm; and -9, -47, 25 mm for auditory, visual, somatosensory, and pain target conditions respectively).

**Figure 5:**
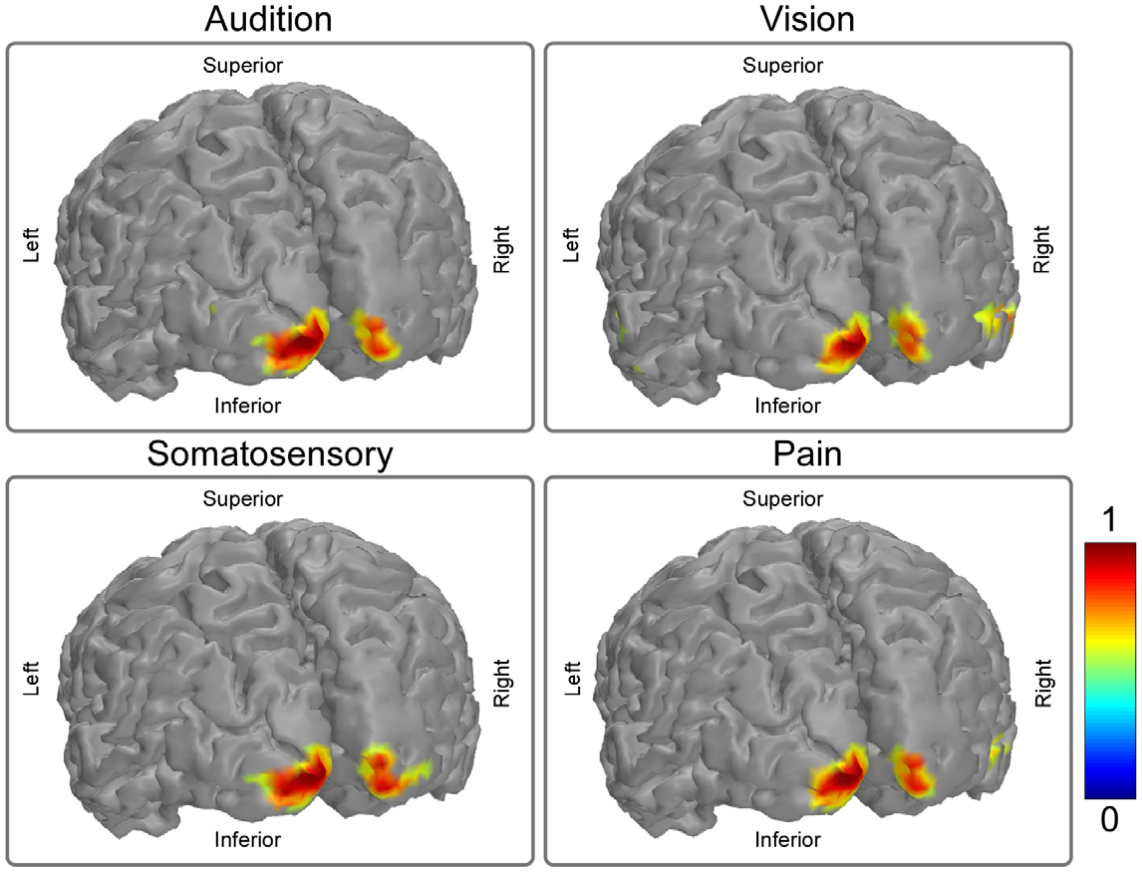
Source localization of α-ERD induced by target stimuli across all sensory modalities. Talairach coordinates (x, y, z) of the sources of the α-ERD are, -9, -99, -7 mm and 16, -95, -12 mm; -6, -99, -5 mm and 14, -96, -4 mm; -6, -99, -5 mm and 17, -97, -3 mm; and -6, -99, -5 mm and 16, -95, -12 mm for auditory, visual, somatosensory, and pain target conditions respectively. Note that the sources of the α-ERD induced by target stimuli across all sensory modalities are similarly located at the bilateral occipital cortices.

Fig. 5 showed the estimated sources of α-ERD induced by auditory, visual, somatosensory, and pain target stimuli. The α-ERD sources were located similarly in the bilateral occipital lobes (Talairach coordinates: -9, -99, -7 mm and 16, -95, -12 mm; -6, -99, -5 mm and 14, -96, -4 mm; -6, -99, -5 mm and 17, -97, -3 mm; -6, -99, -5 mm and 16, -95, -12 mm for auditory, visual, somatosensory, and pain target conditions respectively).

### Effective connectivity analysis

**Figure 6:**
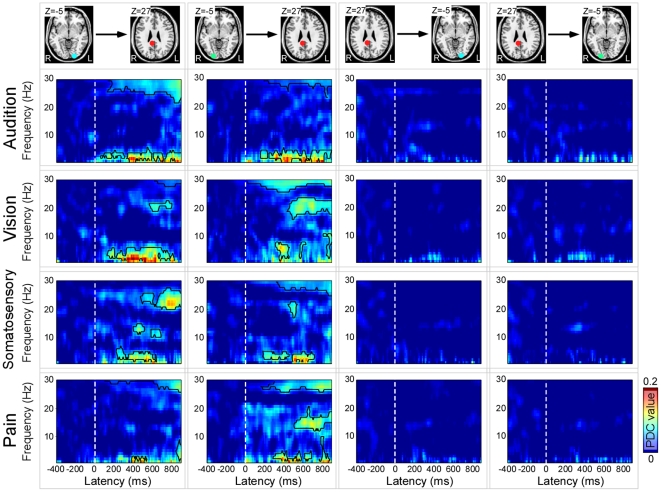
Time-frequency representations of time-varying PDC as a measure of causal influences between the sources of P300 and α-ERD in target conditions across all sensory modalities. *Left panel*: Effective connectivity from bilateral α-ERD sources to P300 sources. Significant increases (marked in black) of effective connectivity from left α-ERD sources to P300 sources could be observed at 96–296 ms and 2–4 Hz, 344–900 ms and 1–4 Hz, and 160–900 ms and 25–30 Hz after auditory target stimuli; at 312–776 ms and 1–6 Hz, 522–784 ms and 19–23 Hz after visual target stimuli; at 216–600 ms and 2–6 Hz, 392–512 ms and 12–15 Hz, 576–680 ms and 10–13 Hz, and 648–900 ms and 20–28 Hz after somatosensory target stimuli; and at 368–552 ms and 2–3 Hz, 824–900 ms and 2–8 Hz, and 600–900 ms and 27–30 Hz after noxious target stimuli. In addition, significant increases (marked in black) of effective connectivity from right α-ERD sources to P300 sources could be observed at 136–900 ms and 2–5 Hz, 232–900 ms and 29–30 Hz after auditory target stimuli; at 344–440 ms and 3–8 Hz, 448–900 ms and 18–24 Hz, and 144–624 ms and 28–30 Hz after visual target stimuli; at 168–712 ms and 2–5 Hz, 448–512 ms and 18–23 Hz, and 336–900 ms and 27–30 Hz after somatosensory target stimuli; and at 288–706 ms and 2–4 Hz, 704–848 ms and 2–10 Hz, 480–900 ms and 12–17 Hz, and 264–900 ms and 27–30 Hz after noxious target stimuli. *Right panel*: Effective connectivity from P300 sources to bilateral α-ERD sources. No significant effective connectivity pattern was observed from P300 sources to bilateral α-ERD sources across all sensory modalities.

In Fig. 6, we displayed the time-frequency regions that exhibited remarkable increase of tvPDC values, which revealed the following findings:

1. For auditory target condition, significant increases in effective connectivity were observed from left side of α-ERD source to P300 source at 96–296 ms and 2–4 Hz, 344–900 ms and 1–4 Hz, and 160–900 ms and 25–30 Hz; and from right side of α-ERD source to P300 source at 136–900 ms and 2–5 Hz, 232–900 ms and 29–30 Hz (Fig. 6).
2. For visual target condition, significant increases in effective connectivity were observed from left side of α-ERD source to P300 source at 312–776 ms and 1–6 Hz, 522–784 ms and 19–23 Hz; and from right side of α-ERD source to P300 source at 344–440 ms and 3–8 Hz, 448–900 ms and 18–24 Hz, and 144–624 ms and 28–30 Hz (Fig. 6).
3. For somatosensory target condition, significant increases in effective connectivity were observed at 216–600 ms and 2–6 Hz, 392–512 ms and 12–15 Hz, 576–680 ms and 10–13 Hz, and 648–900 ms and 20–28 Hz when examining the information flow from left (ipsilateral) side of α-ERD source to P300 source; and at 168–712 ms and 2–5 Hz, 448–512 ms and 18–23 Hz, and 336–900 ms and 27–30 Hz when examining the information flow from right (contralateral) side of α-ERD source to P300 source (Fig. 6).
4. For pain target condition, significant increases in effective connectivity were observed at 368–552 ms and 2–3 Hz, 824–900 ms and 2–8 Hz, and 600–900 ms and 27–30 Hz when examining the information flow from left (ipsilateral) side of α-ERD source to P300 source; and at 288–706 ms and 2–4 Hz, 704–848 ms and 2–10 Hz, 480–900 ms and 12–17 Hz, and 264–900 ms and 27–30 Hz when examining the information flow from right (contralateral) side of α-ERD source to P300 source (Fig. 6).

Across all four sensory modalities, the common region of significant increases in effective connectivity from bilateral α-ERD sources to P300 sources could be consistently observed at about 300–500 ms in latency, and 2–4 Hz in frequency. In contrast, no significant information flow was observed from P300 sources to bilateral α-ERD sources when testing the inverse direction.

## Discussion

In the present study, using oddball task paradigm, task effect (target vs. non-target) on phase-locked ERPs and non phase-locked α-ERD elicited by stimuli of four sensory modalities, i.e., audition, vision, somatosensory, and pain, was assessed. Across the modalities in the target conditions, the scalp topographies and cortical sources were highly similar for P300 and α-ERD across all sensory modalities, and they are respectively located at posterior cingulate cortex and at occipital lobes (Figs. 2, 3, 4, 5). In the non-target conditions, the scalp topographies of α-ERD were maximal distributed at occipital regions for auditory and visual stimuli, but at contralateral central regions for somatosensory and pain stimuli (Fig. 3). These findings implied that P300 and α-ERD in the target conditions were independent of the stimulus modalities, and could mainly reflect the task-related high cognitive activation and attention. In contrast, α-ERD in the non-target conditions was dependent of the stimulus modalities, thus could mainly reflect the sensory perception and judgment. As revealed by effective connectivity, the cortical information was consistently flowed from α-ERD sources to P300 sources in the target conditions (Fig. 6). These findings indicated that P300 in the target conditions was modulated by the changes of α-ERD, which may subserve the basic mechanism of high cognitive information processing in the human brain.

### P300

Several previous studies [5], [7], [8], [9], [10] attempted to find out the location(s) of P300 sources elicited by target stimuli of different modalities (e.g., audition, vision, and somatosensory) using various approaches. Using functional magnetic resonance imaging (fMRI)-constrained ERP source model, Li et al [28] found that the source of P300 elicited by visual stimuli in the landolt ring task was located at the parietal and cingulate cortex. Using fMRI technique, Muller et al [29] observed that the source of P300 evoked by auditory stimuli in the oddball task was mainly located at the parietal and cingulate cortex. Using three-compartment boundary element model in ERP source analysis, Huster et al [9] showed that the most dominant generator of P300 evoked by somatosensory stimuli in their tactile response inhibition task was localized in the posterior mid-cingulate cortex. In addition, other measurement techniques, e.g., intracranial recordings and lesion studies, have been applied in the investigation on the generators of P300, and P300 sources in target conditions were consistently observed in the parietal and cingulate cortex, even though partially conflicting findings were reported across and within methodologies [1].

Similar with most previous studies, we demonstrated that (1) the scalp topographies of P300 elicited by auditory, visual, somatosensory, and pain target stimuli were maximal at parietal regions (Fig. 2), and (2) the main generators of P300 were located at the posterior cingulate cortex (Fig. 4). As the scalp topographies and source locations were remarkably similar for all sensory modalities, we believe that most information expressed by P300 evoked by target stimuli would be *modality independent*, and could mainly reflect the high cognitive activation and attention, which would be common across sensory modalities.

### α-ERD

α-ERD reflected neural rhythm changes of ongoing neural activities at alpha frequency band that were time-locked but not phase-locked to stimulus onset [12], [30]. In previous studies, α-ERD has been consistently observed shortly after the presentation of various types of stimuli, including auditory [15], visual [13], somatosensory [14], and pain stimuli [31]. Apart from these external stimuli, internal mental events can also induce α-ERD, which could thus be believed to play an important role in a variety of cognitive processes [14], [16], [17], [18], [22].

Previously, it has been repeatedly reported that α-ERD was mostly related to primary sensory processing, and α-ERD was showed to be originated from the corresponding sensory cortex [16], [19], [20], [32]. In contrast, Adrian and Matthews [21] provided evidence showing that the origins of α-ERD were the occipital lobes. Similarly, John [22] suggested that alpha rhythm was related primarily to non-specific rather than to specific sensory systems of the brain.

In our study, the scalp topographies and cortical sources of α-ERD induced by target stimuli were highly similarly distributed at the occipital lobes across all sensory modalities (*modality independent*) (Figs. 3 & 5). In contrast, the scalp topographies of α-ERD induced by non-target stimuli were differently distributed, showing a maximal distribution over occipital regions for auditory and visual stimuli, but over contralateral central regions for somatosensory and pain stimuli (*modality dependent*) (Fig. 3). Therefore, it is quite likely that most information expressed by α-ERD induced by the target stimuli was caused by the internal mental events, while α-ERD induced by the non-target stimuli was more related to the external sensory stimuli [31].

### Effective Information flows from α-ERD sources to P300 sources

Previously, α-ERD was reported to coincide with the exogenous ERP components (e.g., P300) [3]. In addition, Yordanova and co-workers [15], [23] investigated the association between P300 and α-ERD in an auditory oddball task experiment. They found that P300 and α-ERD were significantly correlated and manifested similar task effects, thus concluding that α-ERD was functionally associated with P300 elicited by cognitive processing demands[15], [23].

Consistent with previous findings, our results demonstrated that P300 and α-ERD are functionally associated, as the effective connectivity results revealed consistent information flows from bilateral α-ERD sources to P300 sources in target conditions across all sensory modalities, whereas no significant information flows were observed from P300 sources to bilateral α-ERD sources (Fig. 6). With the activation of neural generators of P300, the magnitudes of α-ERD in the target conditions were significantly higher than those in the non-target conditions across the sensory modalities (P<0.001, Fig. 3). In addition, both P300 and α-ERD in the target conditions showed remarkably similar scalp distributions across all sensory modalities (maximum at parietal and occipital regions for P300 and α-ERD respectively). This may imply that both P300 and α-ERD in the target conditions could be influenced by the same cognitive activation, attention, and memory process [3], [18], [19], [32], [33]. The information flows from α-ERD sources (occipital lobes) to P300 generators (posterior cingulate cortex) may be involved in the basic mechanism of high cognitive information communication among these activated regions [3], [34]. Both α-ERD and P300 are long-lasting processes, and α-ERD appears obviously earlier than P300 for all sensory modalities when considering the onsets of these processes (Figs. 2–3). In addition, the majority research of the diffusion tensor imaging (DTI) supported the connectivity pattern between the cingulate cortex and occipital lobes [35].

α rhythm was thought to reflect a spontaneous or ‘idling’ state of human brain [36]. More and more studies indicated that α activities in EEG could be recorded at various scalp locations [37], [38], [39]. Therefore, α rhythm would be related to large ensemble of integrative brain functions, and reflected the function of diffuse and selectively distributed α systems in the brain, giving rise of multiple types of α activities [37], [38], [39]. Being stimulated, the α system, which generated and controlled the α rhythm [40], was able to reset the α activity by changing (reducing or enhancing) and phase-reordering the α oscillations in the post-stimulus interval [36]. α-ERD generated at the occipital regions in the target condition across all sensory modalities was one of the most frequently reported and consistently observed α responses in EEG activities. It is quite likely that, with further processing demands, α-ERD played an active role in network coordination and communication, representing as the effective information flows from occipital lobes (α-ERD sources) to central systems (P300 sources) in this study [41].

Recently, several studies examined the relationships between pre-stimulus α activity and the post-stimulus amplitude of ERPs [42], [43], [44], [45], [46], [47]. An inverse relationship between the pre-stimulus α power and the amplitude of ERPs (the higher the pre-stimulus α power, the lower the amplitude of ERPs) was reported [45], [46], [47]. In contrast, a positive correlation between the pre-stimulus α power and the amplitude of ERPs (especially the P300) was demonstrated [43], [44], [48], [49], [50], [51], [52]. In addition, influences of the phase angle of α activity at stimulus onset over the post-stimulus brain responses were observed [48], [49], [53], [54]. These important findings may be caused by the reason that spontaneous α activity reflected the attentional level and/or mental state of human brain (e.g., large α activity was observed when subjects were at rest), which could influence the subsequent cortical processing (reflected as the post-stimulus ERPs) [16], [38], [55]. The effective information flow from α-ERD sources to P300 generators may reflect such basic neural mechanism, which indicates that the cortical processing (indexed by P300) could be influenced by the attentional level and/or mental state of human brain (indexed by α-ERD).

As we know, the generation of P300 is related to cognitive functioning, e.g., attention allocation and memory updating [1], [3], [33], and α-ERD in the target condition, generated dominantly from occipital lobes, was consistently observed in judgment and memory tasks, which required attention and memory operations [17], [18], [55], [56]. The effective information flow between α-ERD sources and P300 sources, firstly revealed in this study, would also be of great importance in the related cognitive activation and processes. It can be used to help clarify how event-related alpha modulations contribute to cognitive processing and interpret the functional significance of α-ERD. In addition, the combined analysis of time domain ERPs and time-frequency domain EEG oscillations in this context, especially their effective influence, would provide a powerful tool for neuroscientists working in the field of both physiology and psychology to investigate the detail cognitive processing.

In conclusion, our results provided direct evidence for the basic principle of the causality in the association between P300 and α-ERD. In the target condition, the task related cortical information was consistently flowed from α-ERD sources (bilateral occipital lobes) to P300 sources (posterior cingulate cortex) for all four sensory modalities. Thus, the modulation of P300 may be mediated by a cortical-cortical network reflected by the modulation of α-ERD. Such modulations of both P300 and α-ERD may represent physiological/psychological correlates of functions related to attention, state, memory, and task execution in the human brain.

## Materials and Methods

### Subjects

Eighteen right-handed healthy volunteers (nine females), aged from 19 to 29 years (21.8±2.5, mean ± SD), took part in the experiment. All subjects reported normal hearing, normal sensorimotor, and normal or corrected-to-normal vision. All subjects gave written informed consent and were paid for their participation. The procedure was approved by Institutional Review Board of The University of Hong Kong/Hospital Authority Hong Kong West Cluster.

### Stimulation and Experimental Paradigm

#### Stimuli

The auditory stimuli were auditory tones, presented binaurally in a random series at 75 dB SPL through headphones (50 ms plateau, 10 ms rise/fall). The frequency of the tone was either 500 Hz or 1000 Hz. The visual stimuli were center-field presentations (5 cm in height and 5 cm in width) of ‘▴’ and ‘•’ that were viewed from a distance of 130 cm, and lasted for 70 ms. The somatosensory stimuli were square electric pulses of 0.5 ms duration delivered through EEG electrodes to the medial and lateral side of the left hand dorsum. The stimulus intensity was 2 times of the individual somatosensory threshold, and never reported as painful. Noxious stimuli were square electric pulses of 0.5 ms duration delivered through a stainless steel concentric bipolar needle electrode consisting of a needle cathode (length: 0.1 mm, Ø: 0.2 mm) surrounded by a cylindrical anode (Ø: 1.4 mm) [57], [58] to the medial and lateral side of the left hand dorsum. The stimulus intensity was 2 times of the individual perceptual threshold, which was proved to be able to selectively activate the Aδ nociceptive fibers without co-activation of the fast-conducting Aβ fibers [59]. All the noxious stimuli were reported as painful pinprick sensation for all subjects. Note that the nociceptive system was distinct from the non-painful somatosensory system (tactile system) since the nociceptive system projected via Aδ and C nociceptive fibers in the peripheral nerve and via the spinothalamic tract in the anterolateral quadrant of the spinal cord and brainstem [60], while the tactile system projected via Aβ fibers and via the dorsal columns of the spinal cord and the medial lemniscus in the brainstem [60], [61].

#### Procedure

Subjects were seated in a comfortable chair in a lighted shielded room, and were asked to focus their attention on the occurrence of each stimulus. For each stimulus modality (audition, vision, somatosensory, and pain), EEG data were collected from two separated blocks. For one block, both non-target (audition: tones with 500 Hz in frequency; vision: ‘▴’; somatosensory: medial side of the left hand dorsum; pain: medial side of the left hand dorsum) and target stimuli (audition: tones with 1000 Hz in frequency; vision: ‘•’; somatosensory: lateral side of the left hand dorsum; pain: lateral side of the left hand dorsum) were randomly presented with different probabilities (non-target stimuli∶target stimuli = 4∶1). For the other block, the types of non-target and target stimuli were reversed, and they were presented with the same probabilities (non-target stimuli∶target stimuli = 4∶1). Each block consisted of 200 stimuli with inter-stimulus interval (ISI) randomly between 2500 and 3000 ms. The subjects were required to respond as fast and accurate as possible to the predefined target stimuli by pressing the response button upon their appearance, using the right index finger. Reaction times were recorded, and were compared across different sensory modalities using 4-level (audition, vision, somatosensory, and pain) one-way repeated-measures ANOVA with a statistical significance level of P<0.05. Mauchly's test was applied to assess the possible violations of sphericity [62]. If the assumption of sphericity was violated (P<0.05), the degrees of freedom were adjusted (epsilon<0.75: Greenhouse-Geisser correction, epsilon>0.75: Huynh and Feldt correction) [63]. When the main effect of the ANOVA was significant, post hoc pairwise comparisons were performed. The order of the blocks was counterbalanced across subjects. Prior to data collection in each block, the subjects were repeatedly presented with 20 stimuli, to familiarize them with the task.

### EEG recording

The EEG data were recorded using a 64-channel Brain Products system (pass band: 0.01–100 Hz, sampling rate: 500 Hz) using a standard EEG cap based on the extended 10–20 system. The left mastoid was used as the reference channel, and all channel impedances were kept lower than 5 kΩ. To monitor ocular movements and eye blinks, electro-oculographic (EOG) signals were simultaneously recorded from four surface electrodes, one pair placed over the higher and lower eyelid, the other pair placed 1 cm lateral to the outer corner of the left and right orbit.

### EEG data analysis

#### Preprocessing

EEG data were preprocessed using EEGLAB [64], an open source toolbox running under the MATLAB environment. Continuous EEG data were low-pass filtered at 30 Hz. EEG epochs were segmented in 1500 ms time-windows (pre-stimulus 500 ms and post-stimulus 1000 ms), and baseline corrected using the pre-stimulus time interval. Trials contaminated by eye-blinks and movements were corrected using an independent component analysis (ICA) algorithm [64], [65], [66]. In all datasets, individual removed independent components (ICs) had a large EOG channel contribution and a frontal scalp distribution. After ICA and an additional baseline correction, EEG trials were re-referenced to the bilateral mastoid electrodes.

For each subject and each modality (audition, vision, somatosensory, and pain), average waveforms of both target and non-target conditions were computed, time-locked to the onset of the stimulus. Single-subject average waveforms were subsequently averaged to obtain group-level average waveforms. In the target condition, the peak latency and baseline-to-peak amplitude of P300 of each subject were measured at Pz between 300 ms and 600 ms [1], [3]. P300 latency and amplitude across different sensory modalities were compared using 4-level (audition, vision, somatosensory, and pain) one-way repeated-measures ANOVA with a statistical significance level of P<0.05 (the same with the comparison of reaction time). In the non-target condition, the peak latency and baseline-to-peak amplitude of P200 were measured at Cz between 200 ms and 500 ms [67]. The peak latencies of P200 and those of P300 across the sensory modalities were compared using paired sample t-test. The group-level scalp topographies at both P300 and P200 latencies in the target and non-target conditions respectively were computed by spline interpolation for each modality.

#### Time-frequency Analysis

The whole procedure to calculate the magnitude of α-ERD (ER% value) consisted of the following four steps:

1. **Calculation of time-frequency distributions.** Morlet wavelet transform (MWT) was used to estimate the time-frequency distributions (TFDs) of single-trial EEG responses [30] to disclose both phase-locked and non-phase-locked modulations of EEG signal. The parameters of central frequency (ω) and restriction (σ) in MWT were 5 and 0.15 respectively, and TFDs were explored between 1 to 30 Hz in steps of 0.5 Hz.
2. **Baseline correction.** For each estimated frequency, TFDs were baseline corrected using the pre-stimulus interval (-400 to -100 ms), according to the formula: ER(t,f) = [F(t,f)−R(f)]/R(f), where F(t,f) is the signal power at a given time t and at a given frequency f, and R(f) is the signal power of the frequency f averaged within the reference interval [12]. For each subject and each stimulus modality, grand average TFDs were computed for both target and non-target conditions.
3. **Definition of time-frequency region of interest (TF-ROI).** The time-frequency limit of α-ERD (TF-ROI), defined based on previous literature [68], [69], was 8–13 Hz in frequency and 300–800 ms in latency in the target conditions, while it was 8–13 Hz in frequency and 200–700 ms in latency in the non-target conditions.
4. **Measurement of α-ERD magnitude.** Within this TF-ROI, the magnitudes of α-ERD (ER% values) were extracted by computing the mean of the 20% pixels displaying the highest decrease of oscillatory power for each subject at (P3+P4+P5+P6+PO3+PO4)/6 in the target conditions, but at (PO3+PO4+PO7+PO8+O1+O2)/6 for auditory and visual modalities and at (C4+C6+CP4)/3 for somatosensory and pain modalities in the non-target conditions. Note that the choice of the electrodes to measure α-ERD was based on the distribution of scalp topographies, i.e., electrodes displaying the highest decrease of oscillatory power within the defined α-ERD TF-ROI. This “top 20%” summary measure reflected the higher ER% values within the ROI, with the aim of reducing the noise introduced by including all points of the spectrogram, some of which may display little or no response [70].

The magnitudes of α-ERD in the target conditions were compared with those in the non-target conditions across the sensory modalities using paired sample t-test. In addition, α-ERD magnitudes in the target condition across different sensory modalities were compared using 4-level (audition, vision, somatosensory, and pain) one-way repeated-measures ANOVA with a statistical significance level of P<0.05 (the same with the comparison of reaction time). The group-level scalp topographies of α-ERD magnitude within the defined ROI were computed by spline interpolation for each modality in both target and non-target conditions.

#### Source Analysis

For each modality, the locations of the P300 sources were estimated from the group-level average waveforms using the distributed source analysis based on classical LORETA (low resolution brain electromagnetic tomography [71]) analysis recursively applied (CLARA) [72]. In addition, the locations of α-ERD sources were estimated from the group-level averaged TFDs, for each modality, using lead field WMN algorithm [26].

1. **Source estimation of P300 using CLARA.** CLARA is a newly developed iterative distributed source analysis method, and was achieved by performing a weighted LORETA with a reduced source space at each iteration. This iterative approach reduces the blurring of the estimated sources while keeping the advantage of a predefined distributed source model, thus making it easier to determine the location of the source with maximal activity [5], [72]. Singular value decomposition (SVD) regularization with a cutoff of 0.001% and a three iterations scheme was used to perform the CLARA source analysis [25]. The locations and strengths of the regional sources were obtained for a 20-ms long time interval around the latencies of the P300 peaks (audition: 350–370 ms; vision: 350–370 ms; somatosensory: 290–310 ms; pain: 340–360 ms). Source locations were finally transformed to normalized Talairach space.
2. **Source estimation of α-ERD using lead field WMN algorithm.** The cortical current density (CCD) source model was used to solve the inverse problem from the scalp EEG to cortical source distribution using lead field WMN algorithm with the aid of the boundary element model [26], [73]. Tikhonov regularization was applied to minimize errors in EEG inverse solutions. In order to localize the cortical regions that corresponded to the task-related α-ERD, we extracted α-ERD waveforms by averaging 8–13 Hz baseline-corrected spectral power along the frequency axis at each time point, for each subject and each modality. Single-subject average α-ERD waveforms were subsequently averaged to obtain group-level average α-ERD waveforms. With the group-level α-ERD waveforms, the localizations and strengths of the sources estimated by lead field WMN were obtained for the time interval of 300–800 ms (only in the target conditions) [26], [73]. The locations of sources with maximal activities were finally transformed to normalized Talairach space.

#### Effective Connectivity Analysis

The single-trial source waveforms at the estimated sources of P300 and α-ERD in the auditory, visual, somatosensory, and pain target conditions were extracted using eConnectome software [26]. Then ensemble normalization (pointwise subtraction of an ensemble mean and division by ensemble standard deviation) was performed for the single-trial source waveforms, which has been proven to be a critical procedure to dramatically improve the local stationarity of the data [74]. The casual relationship between the sources of P300 and α-ERD was assessed using the time-varying effective connectivity, which is based on the concept of Granger causality [75], and was demonstrated as a powerful capacity for evaluating the direction and strength of causality between neuronal activations [27].

A time-varying multivariate autoregressive (tvMVAR) modeling of the estimated single-trial waveforms was used to reveal the transient effective connectivity. First, the order of tvMVAR model for each subject and stimulus modality was selected based on the information criteria evaluated over a range of model orders [74]. Second, a Kalman smoother, which was proved to provide an accurate estimation of tvMVAR model, was used to identify the tvMVAR coefficients [27]. Third, the connectivity patterns were presented in the time-frequency domain by calculating the time-varying partial directed coherence (tvPDC) for each subject and stimulus modality [76]. Fourth, to test whether the tvPDC values within the post-stimulus interval were significantly different from those within the pre-stimulus interval, a bootstrapping approach [27], which followed the common approaches for testing the significance of time-frequency representations developed in [64] and [77], was adopted. At each time-frequency point to be investigated in the post-stimulus interval, investigated populations and reference populations were collected from the 18 subjects. The null hypothesis was that there was no difference in means between these two populations. Then pseudo-t statistic between the two populations was calculated, and we estimated the probability distribution of the pseudo-t statistic from the reference population by drawing with replacement two populations of the same size. The permutation was executed for 5000 times. The distribution of the pseudo-t statistics from the reference population and the bootstrap P value for the null hypothesis were generated. In such way, the time-frequency regions where the tvPDC values were significantly different relative to the reference interval were detected. Lastly, single-subject tvPDC values were subsequently averaged to obtain group-level average tvPDC values on the time-frequency plane. The EEG source waveforms used for effective connectivity analysis were down sampled at 125 Hz, and the tvPDC values were evaluated from 1 to 30 Hz at a step of 0.5 Hz, and were baseline-corrected by subtracting the average tvPDC values enclosed within the pre-stimulus reference interval (from -350 to -100 ms) at each evaluated frequency.
